# Facile Preparation of Loose P84 Copolyimide/GO Composite Membrane with Excellent Selectivity and Solvent Resistance

**DOI:** 10.3390/polym14071353

**Published:** 2022-03-27

**Authors:** Runlin Han, Kui Wu, Lingfeng Xu

**Affiliations:** School of Chemistry and Chemical Engineering, Jinggangshan University, Ji’an 343009, China; wukui3@mail.sysu.edu.cn (K.W.); linglong707@163.com (L.X.)

**Keywords:** solvent-resistant nanofiltration (SRNF), polyimide membrane, composite membrane, graphene oxide, chitosan

## Abstract

In this study, multilayer graphene oxide (GO) was used to prepare the functional layer of polyimide/GO composite membrane with polyimide (P84) used as the supporting layer. Chitosan added in the functional layer was utilized to adjust the selectivity of the composite membrane. The effects of GO and chitosan contents on membrane morphology and separation performance were investigated in detail. The composite membrane showed high rejection to Congo red and Methyl orange with high flux but low rejection to Na_2_SO_4_ and MgCl_2_ at 0.2 MPa and ambient temperature. The membrane exhibited excellent solvent resistance in *N*,*N*-dimethylacetamide (DMAc) after being crosslinked with 0.5 wt.% triethylene tetramine. The result means that a highly selective and solvent-resistant P84/GO composite membrane was prepared with the facile filtration preparation method.

## 1. Introduction

The development of industrialization has led to environmental problems becoming increasingly more serious. Stricter laws are being introduced all over the world to control environmental pollution. Nanofiltration (NF) is an effective pressure-driven membrane process, introduced in the early 1980s, with pore size below 2 nm. The NF membrane combines low operating pressure and high selectivity, which is widely used in the recovery of metal ions, waste water treatment and pharmaceutical and food industries [1]. The interception of uncharged solute by nanofiltration membrane is mainly based on the mechanism of pore size screening, but the interaction between nanofiltration membrane and solute also needs to be considered, while the separation of charged ions is mainly determined by Donan effect [2]. Organic solvents are often used in chemical and pharmaceutical processes which need to be recovered or separated with solute. However, most of the polymeric membranes are prepared by phase inversion method with membrane materials dissolved in the solvent, which have shown low resistance to strongly polar aprotic solvent. Thus, solvent-resistant nanofiltration (SRNF) has been attracting greater attention in recent decades. Nowadays, the main issues related to the development of SRNF have been the robustness in organic solvents and selectivity [3].

In order to prepare NF membrane with excellent solvent resistance, solvent-resistant or crosslinkable polymeric materials are needed, such as polyacrylonitrile (PAN) [4], polybenzimidazole (PBI) [5], poly(ether ether ketone) (PEEK) [6] and polydimethylsiloxane (PDMS) [7]. PAN membranes are unstable in aprotic solvents, such as dimethyl formamide (DMF) and *N*,*N*-dimethylacetamide (DMAc), which are good common solvents for this polymer. Numerous methods involving crosslinking have been reported to develop PAN membranes with improved solvent stability. The crosslinking of membranes restricts the mobility of the polymer chains and endows the membrane with higher selectivity and permeability in organic solvents [4]. Polyether ether ketone (PEEK) has a rigid aromatic skeleton structure and good resistance to chemical reagents. Hendrix et al. prepared modified PEEK SRNF membranes by the immersion precipitation phase inversion method [8]. The optimized membrane showed the permeation flux of 0.4 L·m^−2^·h^−1^·bar^−1^, while the interception rate of rose Bengal (RB) was 90%. Due to their thermal stability, chemical stability and mechanical strength, polyimides (PIs) have been widely used for membranes in the field of SRNF [9,10,11]. Polyimide can be crosslinked by diamine at low temperature, and its membrane forming and mechanical properties are outstanding. It gradually becomes one of the most studied membrane materials in the field of SRNF membrane [12]. Lenzing P84 polyimide have been crosslinked with aliphatic diamines to prepare solvent stable organic solvent nanofiltration membranes. The integrally skinned membranes were stable in many organic solvents, including toluene, methanol, methylene chloride, tetrahydrofuran, dimethyl formamide and n-methyl pyrrolidone, which provided possibilities for applications in harsh solvent environments [13,14].

Graphene oxide (GO) is a well-known two-dimensional material with polar functional groups such as epoxide, hydroxyl and carboxy groups, which have emerged as separation membrane material with unique layered structures [15]. GO-based membranes have attracted extensive attention for their excellent molecular separation performance, especially NF performance, because they have a stratified structure, which provides suitable ion channels and enhances the selectivity of NF membrane [16,17,18]. To realize GO composite membrane, vacuum filtration has been used as one of simplest routes. Flow-directed assembly has the advantage of controlling the thickness of the film. Dikin et al. [19] used this method with ease to control the membrane thickness from 1 to 30 μm. However, most of the 2D membranes will be compacted during filtration at elevated pressure. It is necessary to adjust the interlayer spacing with nanomaterial [20,21]. It has been found that GO with functional groups can chemically react with the amine group of chitosan and form stable covalent bonds between the inorganic material and polymer, which is beneficial to the mechanical and chemical stability of the membrane [22]. Chitosan composite membranes embedded with GO have shown excellent stability in the pervaporation of the pre-esterification process [23]. Hence, GO and chitosan are both potential solvent-resistant membrane materials.

In the present study, P84 membrane was used as the support for the composite membrane while GO membrane was used as the functional layer. We utilized the solvent-resistant chitosan additive to adjust the spacing and improve the performance of the 2D GO membranes. The membranes were crosslinked with triethylenetetramine (TETA) to improve the solvent resistance. The morphology of the membranes were characterized by scanning electron microscope (SEM) and atomic force microscope (AFM) while the solvent stability was tested with membrane immersed in DMAc solution for a certain time. The membrane showed high permselectivity and flux at 0.2 MPa with high solvent resistance in DMAc immersion.

## 2. Experimental

### 2.1. Materials and Instruments

P84 (HP Polymer Inc., Vienna, Austria, Mw: 25,000) was used to prepare the supporting membrane while GO (Tanfeng Tech Inc., Suzhou, China) was used as the functional layer material. The flake size of GO was about 10–50 μm and there were about 6–8 layers. TETA was introduced to crosslink the polymer chain to improve the solvent resistance in composite membrane. Chitosan, DMAc and other chemicals used in the experiments were all analytical purity grade without further purification. The separation performance of the composite membrane was tested with a flat sheet dead-end filtration set-up. The dye concentration was analyzed with UV-Vis Spectrometer (721, Shanghai Youke, Shanghai, China) and the salt concentration was studied with a conductivity meter. In order to decrease the concentration polarization, a stirring bar was fixed above the membrane surface and a magnetic stirrer (Jiangsu Jiangyin Science Research Instrument Plant, Jiangyin, China) was installed below the membrane set-up with a speed of 700 r/min.

### 2.2. Membrane Preparation

The supporting polyimide membranes used in this work ware prepared in our laboratory with a phase inversion method as follows. DMAc (Sinopharm, Shanghai, China) was used as solvent to prepare 15% polyimide solution, and the solution was stirred for 12 h to obtain a homogeneous solution. The membrane was fabricated on a horizontal non-woven polyester fabric with a glass blade. After evaporation in air for 5 s, the membrane was immersed in the coagulation water bath with a temperature of about 20 °C. The prepared polyimide membrane was soaked in water for more than 24 h to completely remove the solvent for later use. In the P84/GO composite membrane preparation, the prepared polyimide membrane was used as the supporting membrane. A certain amount of GO with or without additive was added in the 1% acetic acid/water solution and filtered by the polyimide membrane with an area of 41 cm^2^. The formed membrane was immersed in a 0.5 wt. % TETA/water solution at 60 °C and 3 min for crosslinking. Then the membrane was removed and cleaned with de-ionized water for further characterization.

### 2.3. Membrane Characterization

**Figure 1:**
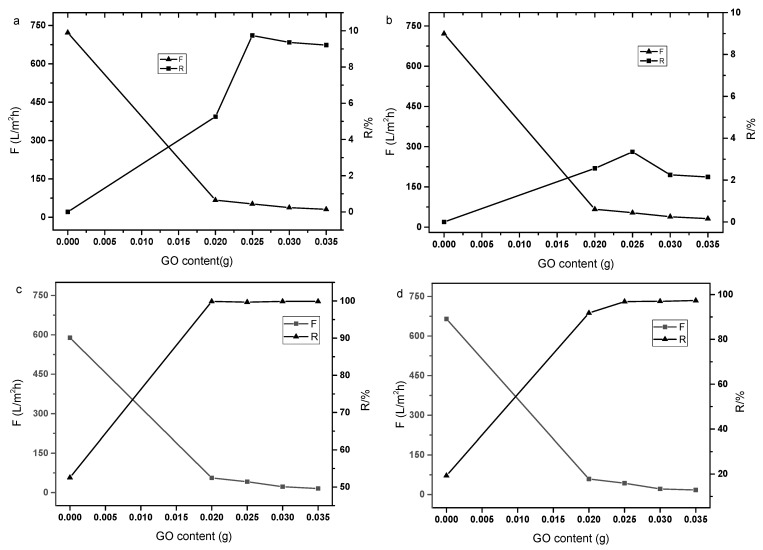
Effect of GO content in the solution on the performance of composite membrane ((**a**): Na_2_SO_4_, 1 g·L^−1^; (**b**): MgCl_2_, 1 g·L^−1^; (**c**): Congo red, 50 mg·L^−1^; (**d**): Methyl orange, 50 mg·L^−1^).

The morphology of the membrane was analyzed by SEM (FEI Nova NanoSEM 450, Hillsboro, OR, USA) and AFM (Bruker, Dimension ICON, Karlsruhe, Germany). The samples were sputtered with gold after they were immersed in the ethanol solution to observe the SEM images of the membrane. The performances including flux (F) and rejection (R) of the membrane were characterized by the dead-end membrane set-up. The membranes were pressed under 0.4 MPa for 30 min in order to obtain stabilized membrane performance. The concentrations of dye solution including Congo red and Methyl orange solution were all fixed 0.05 g·L^−1^ while the concentration of inorganic salts are fixed 1 g·L^−1^. Membrane performances including pure water flux and rejection to solutes were measured under the pressure of 0.2 MPa at 20 °C. The permeation flux (*F*) was calculated as follows:

(1) $$F=\frac{W}{At}$$

where *W* is the total volume of the water or solution permeated during filtration process; *A* is the valid membrane area; and *t* is the operation time. Rejection, *R*, was calculated using the following equation:

(2) $$R=(1-\frac{C_{p}}{C_{f}})\%$$

where *C_p_* and *C_f_* are the concentrations of the permeate solution and the feed solution, respectively. All the experiments on flux and rejection were repeated three times with the average data shown in the Figure 1.

## 3. Results and Discussion

### 3.1. Effect of GO Content in the Solution on the Performance of Composite Membranes

The separation performance of pure P84/GO laminate composite membrane with different GO mass on P84 base membrane was tested. The GO mass of the composite membrane was 0.02 g, 0.025 g, 0.03 g and 0.035 g, respectively. The separation performance of the composite membrane on inorganic salt Na_2_SO_4_, MgCl_2_, Congo red and Methyl orange were studied. Figure 1 shows the separation performance of P84/GO laminate composite membrane. It can be seen from the figure that the P84 support membrane has no rejection performance for inorganic salts Na_2_SO_4_ and MgCl_2_, and low rejection rate for dyes. The support membrane showed high flux (about 700 L·m^−2^·h^−1^) at 0.2 MPa and ambient temperature. As shown in Figure 1a, the membrane rejection increased with the GO content while the membrane flux decreased gradually. When the GO content was 0.025 g, the separation performance of the composite membrane was stable. The P84/GO laminate composite membrane had a rejection of Na_2_SO_4_ below 10%. Figure 1b shows the MgCl_2_ separation performance of the composite membrane. Similar characteristics were observed while the rejection to MgCl_2_ was less than 4%. The lower rejection to divalent cation may have been caused by the electrostatic repulsion of the active groups in the GO layer. The basal planes of the GO nanosheets contain hydroxyl and epoxide groups while their edges contain ketone and carboxylic acid groups, which makes the membrane negatively charged in the surface [24], although the PI supporting membrane crosslinked with molecules with abundant amino groups are often positively charged [25]. The P84/GO laminate composite membrane was observed to have high rejections to Congo red and methyl orange. As shown in Figure 1c, with the increase in the GO content, the membrane rejection to Congo red clearly increased from 53% to 99.9% while the flux decreased from 589 to 15.3 L·m^−2^·h^−1^. The rejection to Methyl orange dye also increased to 97% exhibiting a similar flux decline phenomenon, as shown in Figure 1d. Continued increases in GO content higher than 0.025 g did not increase the rejection of ions, which may have been caused by low permeation of water molecules. The higher rejection to Congo red dye may have been caused by the higher molecular weight (697 Da) compared with that of Methyl orange (327 Da). The loose membrane showed obvious molecular sieve effect on the organic dyes. In order to maintain suitable flux and high rejection of the composite membrane, the GO content was fixed 0.025 g in the subsequent experiments.

### 3.2. Effect of Chitosan Content on the Membrane Performance

**Figure 2:**
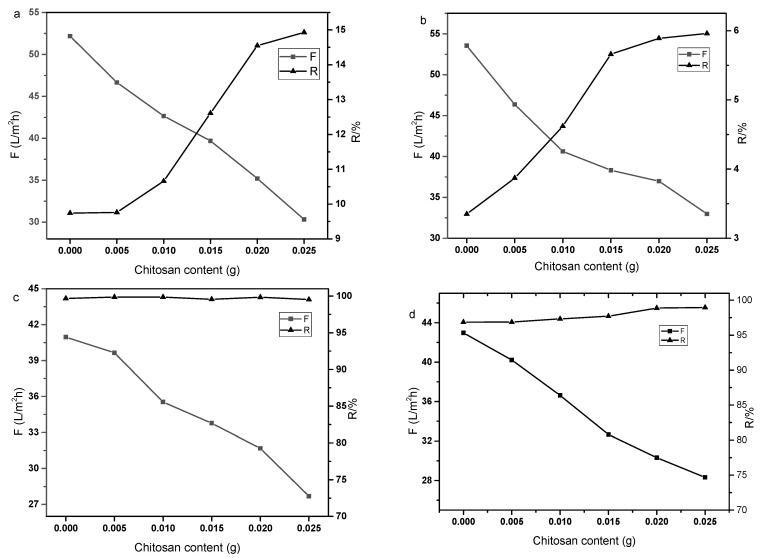
Effect of chitosan content on the membrane performance ((**a**): Na_2_SO_4_, 1 g·L^−1^; (**b**): MgCl_2_, 1 g·L^−1^; (**c**): Congo red, 50 mg·L^−1^; (**d**): Methyl orange, 50 mg·L^−1^).

There are abundant active amino and hydroxyl groups in the main chain of chitosan which can react with the active groups of GO. When treated at elevated temperature with GO, the epoxy group on the GO sheet can react with the primary amino group in the chitosan chain. The abundant covalent bond between the polymer and GO can form a crosslinked structure, which improves the stability of the GO membrane. In addition, the amino group in the chitosan molecule can enhance the electrostatic interaction with ions in the feed solution and adjust the layer space of GO which control the separation performance of the composite membrane easily. Figure 2 shows the performance of composite membranes prepared with different chitosan contents. The weight of chitosan added were 0.005 g, 0.01 g, 0.015 g, 0.02 g, 0.025 g, respectively. The rejection and flux of the composite membrane to Na_2_SO_4_, MgCl_2_ solutions, Congo red and methyl orange solutions were tested. As can be seen from the figure, with the increase in the added chitosan content, the selectivity of the composite membrane was improved to some extent. The flux of the membrane decreased from about 55 to 30 L·m^−2^·h^−1^ and the rejections of Na_2_SO_4_ and MgCl_2_ were 15% and 6%, respectively. The rejection rates of Methyl orange and Congo red were all above 99%, but the flux of the membrane decreased with the increase in chitosan mass. It can be seen from the test results that adding chitosan to the GO layer can improve the interception performance of the composite membrane to some extent.

### 3.3. Morphology of the P84/GO Composite Membranes

**Figure 3:**
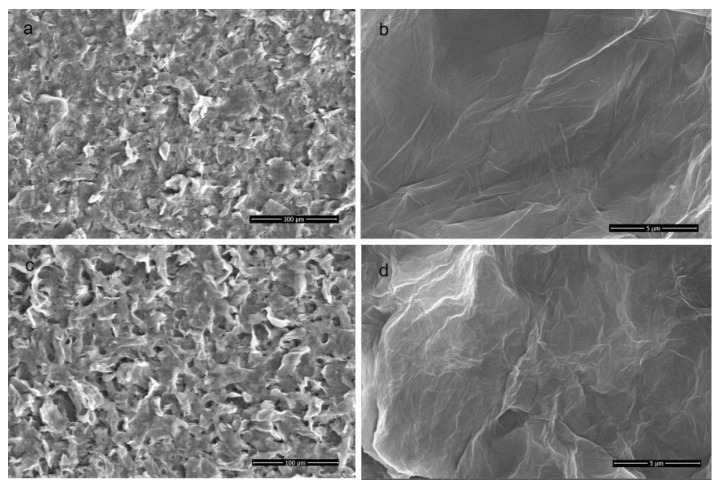
SEM images of the top surface of membranes with different chitosan content ((**a**,**b**): pure GO membrane; (**c**,**d**) GO membrane with chitosan).

**Figure 4:**
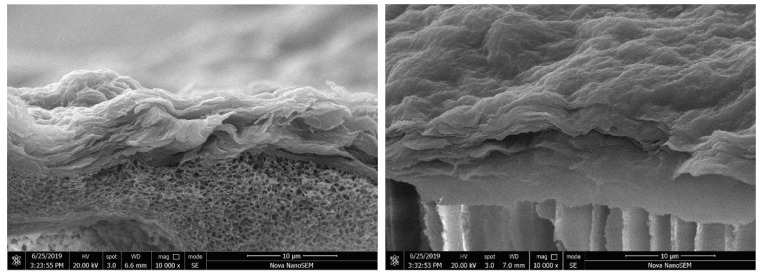
Cross-section morphology of P84/GO composite membranes (**left**, without chitosan, **right**, with chitosan).

The membrane morphology was characterized by SEM and AFM. It can clearly be observed from Figure 3 that the P84/GO composite membranes with different magnifications all showed dense and smooth surfaces. Figure 3a,b shows morphologies of the pure GO membrane with different magnifications. The scale bars are 100 and 5 μm, respectively. The rough surface observed on the top surface shown in Figure 3a suggests that the deposition of GO on the P84 membrane was enough to completely cover the support top surface of P84 membrane. No defect could be observed in the high resolution picture as shown in Figure 3b, which interprets the high rejections to organic dyes with relatively low molecular weights. The membrane surface became coarser and more protrusions were observed on the membrane surface with 0.02 g chitosan involved, as shown in Figure 3c,d. The membranes had no defect which endowed the membranes with high selectivity, but the ions can easily transport though the layer of the GO in the functional layer. Figure 4 is the cross-section view of the P84/GO composite membrane with chitosan added. The cross-sectional images of composite membrane showed significant boundary between support and coating layer because the functional layer of GO had a layered structure while the supporting layer of P84 membrane often showed finger-like pores in the cross-section images [12]. It can be observed from the image that the lower part of the figure is the base membrane with a typical asymmetric microstructure, and the upper part is a clear dense GO membrane with layered structure. Dense and layered GO separation layers can be clearly observed in the figure. The thickness of the functional layer was very low (about 10 μm), as shown in Figure 4, which endowed the membrane with high water flux at low pressure. After chitosan was added, the thickness of the functional layer increased to some extent.

**Figure 5:**
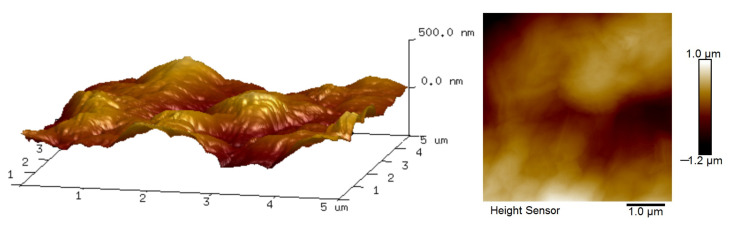
3D AFM morphology of P84/GO composite membrane with chitosan.

Figure 5 shows the surface roughness of the P84/GO composite membrane with chitosan; the membrane was characterized by AFM with the testing area fixed 5 μm × 5 μm. As can be seen from the image, the membrane has a relatively rough surface, which is consistent with the SEM images of the composite membrane. The formed rough surface may have been caused by the pores in the P84 membrane and filtration pressure during composite membrane preparation.

### 3.4. Solvent Resistance in P84/GO Nanofiltration Membrane

**Figure 6:**
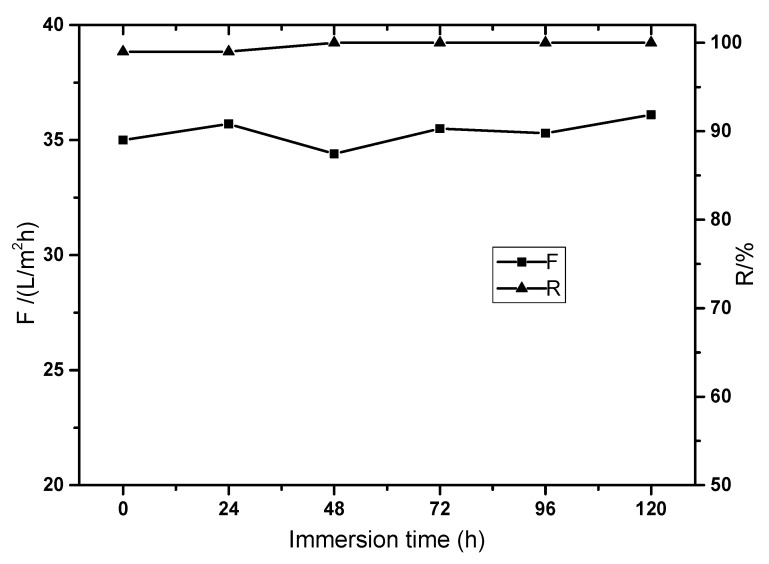
The solvent resistance in the P84/GO composite membrane.

Crosslinked PI membrane often has excellent solvent resistance. In order to test the solvent resistance in the composite membrane crosslinked with TETA, the membrane was immersed in DMAc solution for 120 h and tested every 24 h. The membrane rejection to Methyl orange was still above 99% while the membrane flux did not decrease during static immersion, as shown in Figure 6, which implies excellent solvent resistance in the composite membrane. The effective crosslinking was constructed with TETA and P84 membrane while the GO and chitosan layer were originally stable in DMAc solution. The membrane flux did not decline because the main mass transfer resistance was in the functional layer and the space of the GO layer did not change during immersion in solvent because the polymer additive chitosan was not dissolved in static DMAc solution.

## 4. Conclusions

P84/GO composite membrane was successfully prepared with a facile filtration preparation method. The P84/GO composite membrane was found to have excellent permselectivity with GO and chitosan used as the functional layer. At 0.2 MPa and ambient temperature, its rejection to Methyl orange and Congo red were all above 99% with high flux above 30 L·m^−2^·h^−1^. The rejections to inorganic salts Na_2_SO_4_ and MgCl_2_ were relatively low compared with organic dye molecules. The loose composite NF membrane showed potential application in dye and salt separation. With the increase in chitosan content, the membrane showed a denser and rougher functional layer with improved rejection and decreased water flux. After crosslinking with TETA at 60 °C for 3 min, the membrane showed excellent solvent resistance. During static immersion in pure solvent DMAc for 120 h, the rejection to Methyl orange and flux did not decline with operation time. This demonstrates that the full crosslinking of the supporting layer and the membrane can be used in the SRNF process.

## Data Availability

The data presented in this study are available on request from the corresponding author.
